# Graft Failure in Patients With Hematological Malignancies: A Successful Salvage With a Second Transplantation From a Different Haploidentical Donor

**DOI:** 10.3389/fmed.2021.604085

**Published:** 2021-06-04

**Authors:** Yu-Qian Sun, Yu Wang, Feng-Rong Wang, Chen-Hua Yan, Yi-Fei Cheng, Yu-Hong Chen, Yuan-Yuan Zhang, Ting-Ting Han, Wei Han, Pan Suo, Lan-Ping Xu, Xiao-Hui Zhang, Kai-Yan Liu, Xiao-Jun Huang

**Affiliations:** Beijing Key Laboratory of Hematopoietic Stem Cell Transplantation, National Clinical Research Center for Hematologic Disease, Peking University People's Hospital, Peking University Institute of Hematology, Beijing, China

**Keywords:** graft failure, second transplantation, cyclophosphamide, fludarabine, haploidentical

## Abstract

Graft failure (GF) is a fatal complication of allogeneic stem cell transplantation, especially after haploidentical transplantation. The mortality of GF is nearly 100% without an effective salvage method. A second transplantation is usually necessary to save the patient's life. However, there is no standardized regimen, and the outcome is usually disappointing. We report on a prospective single-center study using a reduced-intensity conditioning regimen with different haploidentical donors (HIDs). Patients with GF after the first transplantation were enrolled in a prospective single-arm clinical trial (ClinicalTrials.Gov ID: NCT03717545) at the Peking University Institute of Hematology. The conditioning regimen consisted of fludarabine (30 mg/m^2^) (days−6 to−2) and cyclophosphamide (1,000 mg/m^2^/day) (days−5 to−4). Patients underwent a second transplant from a different HID using a granulocyte colony-stimulating factor primed bone marrow and peripheral blood stem cells. The primary outcome was neutrophil engraftment at day 28. The secondary outcomes included platelet engraftment at day 100, transplant-related mortality (TRM) at day 30, TRM at day 100, and overall survival (OS) at 1 year. From March 2018 to June 2020, 13 patients were enrolled in this clinical trial. Of the 13 patients, five had acute myeloid leukemia, five had acute lymphoblastic leukemia, two had myelodysplastic syndromes, and one had a non-Hodgkin lymphoma. The median age at first transplantation was 38 years (range, 8–55 years). As for the first transplantation, 11 patients underwent haploidentical transplantations and two underwent unrelated donor transplantations. At the time of GF, three patients had complete donor chimerism, five had mixed chimerism, and five had complete recipient chimerism. The median time from the first transplantation to the second transplantation was 49 (range 35–120) days. The medians of infused cell doses were as follows: mononuclear cells 7.93 (5.95–12.51) × 10^8^/kg and CD34 + cells 2.28 (0.75–5.57) × 10^6^/kg. All 13 patients achieved neutrophil engraftment after the second transplantation, with a median engraftment time of 11 (range 10–20) days after transplantation. The platelet engraftment rate on day 100 after transplantation was 76.9%. The TRMs at day 30, day 100, and 1-year were 0, 0, and 23.1%, respectively. The OS and disease-free survival at 1-year were 56.6 and 48.4%, respectively. For patients with GF after first transplantation, a second transplantation using a fludarabine/cyclophosphamide regimen from a different HID was a promising salvage option. Further investigation is needed to confirm the suitability of this method.

## Introduction

Graft failure (GF) is defined as the failure to achieve sustained engraftment following allogeneic stem cell transplantation (allo-SCT). It is a fatal complication of allo-SCT and is associated with considerable morbidity and mortality, most notably infections and hemorrhagic complications due to marrow hypoplasia. The occurrence of GF is associated with several factors, including underlying disease, human leukocyte antigen disparity, conditioning regimen, graft, cell counts, and donor-specific antibodies (DSA) (1). While the incidence of GF is <5% in general, it is more frequent in patients after haploidentical stem cell transplantation, where the incidence is around 10% in T cell-depleted modality (2), 13% in post-transplant cyclophosphamide-based T cell depleted modality (3), and 1% in the Beijing protocol (4). Although it is a rare complication, the mortality of GF is almost 100% without salvage therapy (5).

While second transplantation is almost the only salvage therapy available, the prognosis after second transplantation is still very poor, and the overall survival (OS) reported in retrospective studies was 11–37% (6–8). A possible reason for this may include the high treatment-related mortality. It is therefore important to develop a better method for second transplantation with a safe conditioning system that can ensure successful engraftment and avoid early toxicity. However, there is currently no consensus on protocols for second transplantations after GF. Herein, we report on a prospective single-center study using a reduced-intensity conditioning regimen with different haploidentical donors (HIDs).

## Materials and Methods

### Study Design

This prospective, single-arm clinical trial was performed at the Peking University Institute of Hematology, China. This study was approved by the ethics committee of Peking University People's Hospital. All patients provided written informed consent before enrollment. The study was registered as a clinical trial (ClinicalTrials.Gov: NCT03717545). The inclusion criteria were as follows: (1) diagnosis of acute leukemia, myelodysplastic syndromes, or lymphoma; (2) graft failure after the first allogeneic stem cell transplantation; and (3) signed informed consent to the current study. Patients will be excluded if with any of the following criteria: (1)uncontrolled active infection; (2)unctrolled active GVHD; (3)significance organ dysfunction: serum total bilirubin≥2ULN or serum creatine ≥1.5 upper limit of normal (ULN), or ejection fraction <50%, or symptomatic heart failure; (4)poor performance (ECOG >2); (5)expected life <28 days; (6) patient can not cooperate the treatment; (7)the physician evaluated as not suitable. From March 1, 2018 to April 30, 2020, a total of 13 patients were enrolled in this study. The last follow-up date was July 31, 2020.

### Protocol for the Second Transplantation

The conditioning regimen consisted of the following agents: fludarabine (30 mg/m^2^/day, injected i.v.) from day−6 to day−2; cyclophosphamide (1,000 mg/m^2^/day, injected i.v.) on days−5 and−4. A different HID was selected for the second transplantation. The donor was administered with granulocyte colony-stimulating factor (G-CSF) 5 ug/kg/d from day−3 to the end of collection. All of the recipients except one (due to coronavirus disease, the planned bone marrow collection was canceled) were administered G-CSF-mobilized bone marrow and peripheral blood stem cells (PBSC). Bone marrow was harvested on day 1, and PBSC was harvested on day 2. Graft-vs.-host disease (GVHD) prevention consisted of basixilimab 20 mg on days −1 and + 4, plus cyclosporine A (trough concentration 150–250 ng/ml), and mycophenolate mofetil. The infection prophylaxis was based on our protocol as previously literature (4). Cytomegalovirus (CMV) and Epstein-Barr virus (EBV) were monitored twice per week using real-time polymerase chain reaction. Hematopoietic chimerism was evaluated using fluorescent *in situ* hybridization for sex-unmatched pairs or using the short tandem repeat technique.

### Definitions

The primary endpoint was neutrophil engraftment within 28 days after the second transplantation. The secondary endpoints were platelet engraftment, acute GVHD (aGVHD), chronic GVHD (cGVHD), CMV reactivation, EBV reactivation, relapse, treatment-related mortality (TRM), OS, and disease-free survival.

Neutrophil engraftment was defined as the first of three consecutive days with an absolute neutrophil count ≥0.5 × 10^9^/L. Platelet engraftment was defined as the first of seven consecutive days with a platelet count ≥20 × 10^9^/L without transfusion dependence. Primary GF was defined as the failure to surpass a threshold absolute neutrophil count of 0.5 × 10^9^/L by day 28 after transplantation. Secondary graft failure was defined as subsequent cytopenia of at least two lines (i.e., neutrophil decline to < 0.5 × 10^9^/L, platelet count decline to <20 × 10^9^/L) after initial engraftment. Complete donor chimerism was defined as the presence of at least 95% donor hematopoietic cells. aGVHD and cGVHD were graded according to previous criteria (9, 10). OS was defined as the time from the first day of transplantation to the time of death as a result of any cause. Follow-up data for survival were recorded when the patient was last verified to be alive. Disease-free survival **(**DFS) was defined as the time from transplantation to the time of relapse, disease progression, or death, whichever occurred first. Relapse was defined as the reappearance of blasts in the blood, bone marrow (**>**5%), or any extramedullary site after complete remission. TRM was defined as any cause of death other than relapse. The hematopoietic cell transplantation-specific comorbidity index score was evaluated according to the literature (11).

### Statistical Analyses

Continuous variables were represented as medians, and categorical variables were represented as percentages. OS and DFS were estimated using the Kaplan–Meier method. The cumulative incidences of engraftment, GVHD, treatment-related mortality (TRM), and relapse were estimated using a competing risk model. Death and relapse without developing GVHD were treated as competing events for GVHD, whereas relapse and TRM were treated as events competing with each other. A *p* < 0.05 for a two-sided test was considered to be significant. The multivariate Cox proportional model and survival analysis were calculated with SPSS software (SPSS 16.0, Chicago, IL, USA). The cumulative incidence was calculated with R statistical software, version 3.6.0 (R Foundation for Statistical Computing, Vienna, Austria).

## Results

### Patient Characteristics

From March 1, 2018 to April 30, 2020, a total of 13 patients were enrolled in this study. The last follow-up date was July 31, 2020. Detailed information on demographic information, disease information, and first transplantation are summarized in Table 1. At the time of the first transplantation, six patients were DSA positive, among which there were four patients with mean fluorescence intensity >5,000, who were administered rituximab and received third-party cord blood (CB) transplants according to our protocol (12).

**Table 1:** Patient characteristics at first transplantation.

**Variables**	**Value**
Age (years), median (range)	37 (8–55)
Gender, man (%)	5 (38.5%)
Underlying disease (status)	
AML (CR1/CR2)	5 (5/0)
ALL (CR1/CR2)	5 (3/2)
MDS (EB1)	2
NHL	1
DSA-MFI	
0	7
0–2,000	1
2,000–5,000	1
5,000-10,000	4
Donor type	
Unrelated donor	2
Haploidentical donor	11
Donor gender (male/female) (2 unknown)	9/2
Donor ABO (match/ major mismatch/ minor mismatch/ bidirectional mismatch)	5/4/3/1
Conditioning regimen	
Bu-based	12
TBI-based	1
First graft	
BM + PB	11
PB	2
Third-party CB*	2
MNC (×10^8^/kg), median (range)	8.46 (5.68–24.5)
CD34+ cell dose (×10^6^/kg), median (range)	1.54 (0.64–9.28)

*AML, acute myeloid leukemia; ALL, acute lymphoblastic leukemia; MDS(EB1), myelodysplastic syndrome with excessive blast−1; NHL, non-Hodgkin lymphoma; CR1, first complete remission; CR2, second complete remission; DSA, donor specific antibodies; MFI, median fluorescence intensity; Bu, busulfan; TBI, total body irradiation; BM, bone marrow; PB, peripheral blood; CB, cord blood; MNC, mononuclear cell*.

At the time of the second transplantation, 12 were primary GF cases, and one was a secondary GF case (Table 2). At the diagnosis of GF, three cases were identified as having complete donor chimerism, four had mixed chimerism, and six had complete recipient chimerism. The median time from the time of first transplantation to second transplantation was 42 days (range, 33–120 days). For the second transplantation, the median donor age was 47 (10–69) years.

**Table 2:** Summary of the second transplantation.

**Variables**	**Value**
GF type	
Primary	12
Secondary	1
Chimerism	
Complete donor	3
Mixed	4
Completed recipient	6
Time from the first to second transplant (days)	42 (33–120)
Second donor	
Donor age (years)	47 (10–69)
Donor gender, man (%)	5
Donor relation (parents/sibling/child/other)	6/4/2/1
DSA positive	1
Second graft	
BM + PB	12
PB	1
Third-party CB	1
MNC	9.44 (5.95–12.51)
CD34+	1.76 (0.75–5.57)
Engraftment	
Neutrophil	13 (10–21)
Platelet	16(9–78)
Grade 2-4 Agvhd	44.4%
1-year TRM	23.1%
1-year relapse	23.1%
1-year DFS	48.4%
1-year OS	56.6%

*GF, graft failure; DSA, donor specific antibodies; BM, bone marrow; PB, peripheral blood; CB, cord blood; MNC, mononuclear cell; aGVHD, acute graft vs. host disease; TRM, treatment-related mortality; DFS, disease-free survival; OS, overall survival*.

### Outcomes After the Second Transplantation

#### Engraftment

**Figure 1:**
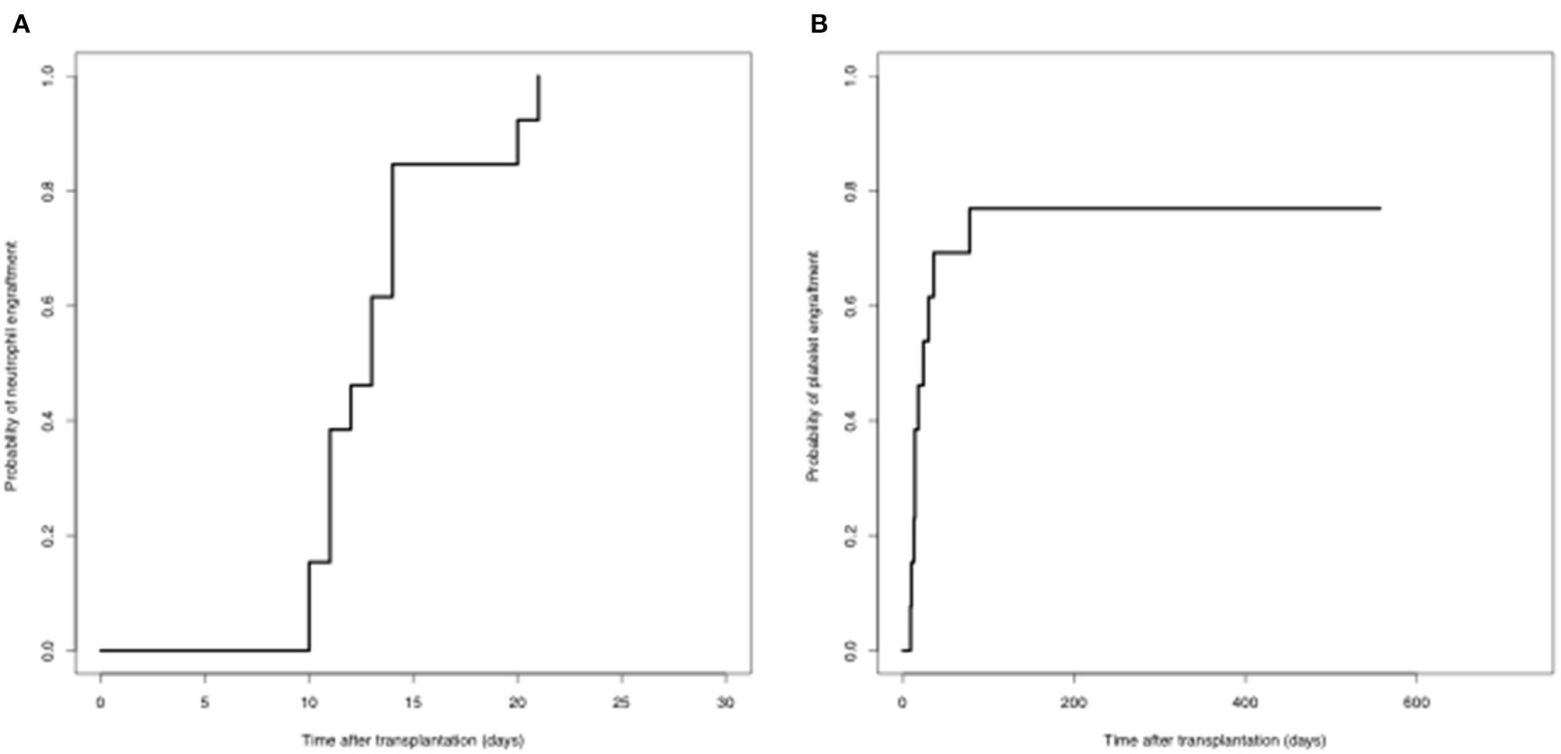
Engraftment **(A)** neutrophil engraftment; **(B)** platelet engraftment.

All patients achieved complete donor chimerism after the second transplantation. The median time of neutrophil engraftment was 13 (range, 10–21) days after the second transplantation. Ten out of 13 (76.9%) patients achieved platelet engraftment at the last follow-up. The median time of platelet engraftment was 16 (range, 9-78) days after the second transplantation (Figure 1).

#### GVHD

**Figure 2:**
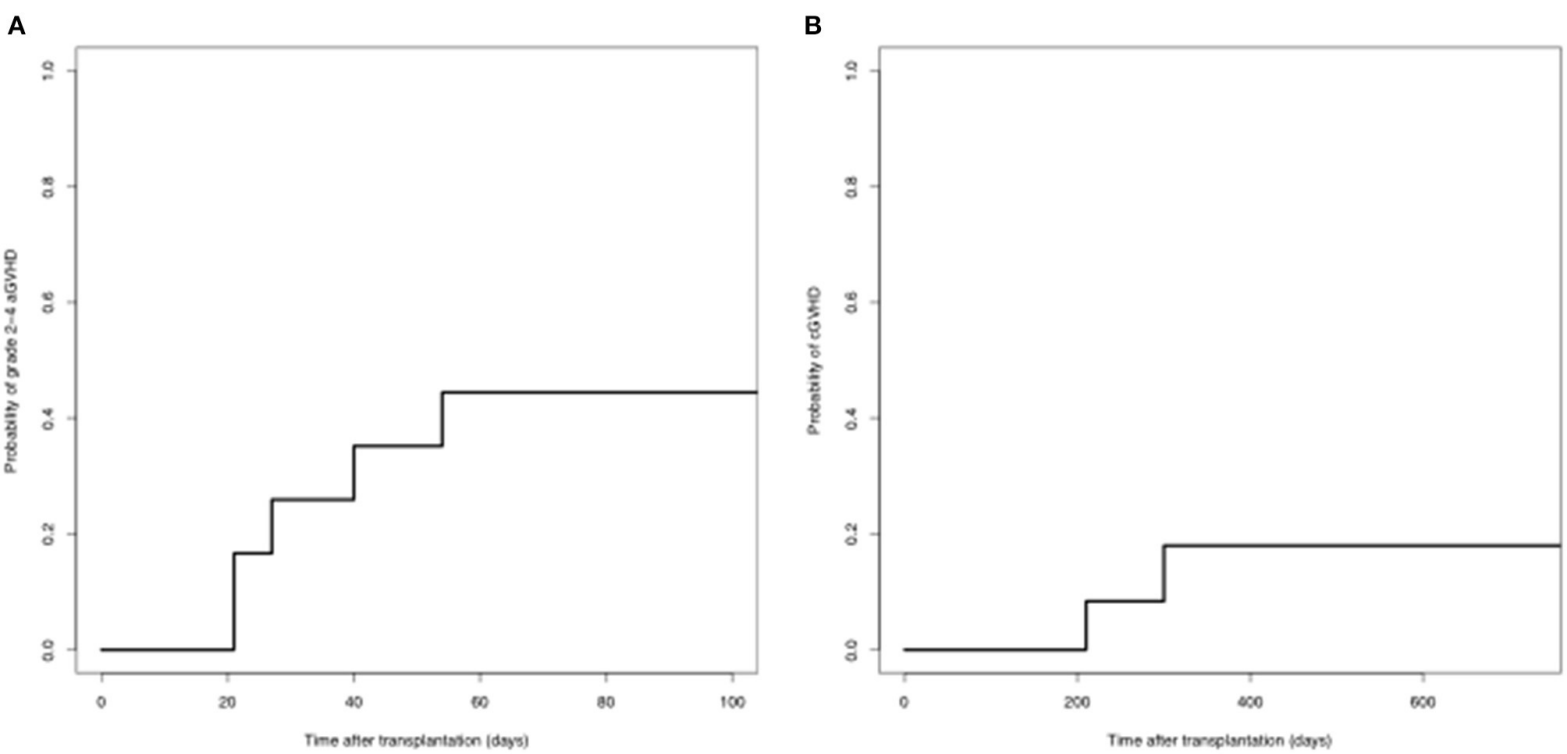
GVHD after second transplantation **(A)** grade 2-4 aGVHD; **(B)** chronic GVHD.

Nine patients developed aGVHD, of which three were grade 1, 3 were grade 2, and 3 were grade 3-4. The cumulative incidence of grades 2-4 aGVHD on day 100 was 44.4%. Eleven patients were evaluated for cGVHD. Two patients developed cGVHD. The cumulative incidence of cGVHD at 1 year was 17.9% (Figure 2).

#### TRM

None of the patients died within 30 days after the second transplantation. At the last follow-up, four patients died due to transplant complications, two were due to severe infection, and t were due to severe GVHD. The cumulative incidence of TRM at 30 days, 100 days, 180 days, and 1 year was 0, 0, 15.3, and 23.1%, respectively.

#### Infection

Eleven patients developed CMV reactivation. The cumulative incidence of CMV reactivation on day 100 was 84.6%. Only one patient developed EBV reactivation 14 days after transplantation.

#### Relapse

At the last follow-up, three patients relapsed at 72, 93, and 273 days after transplantation, respectively. The cumulative incidence of relapse after 1 year was 23.1%. Among the three relapses, two died without any further treatment, and one survived without disease after undergoing chimeric antigen receptor T**-**cell therapy.

#### OS and DFS

**Figure 3:**
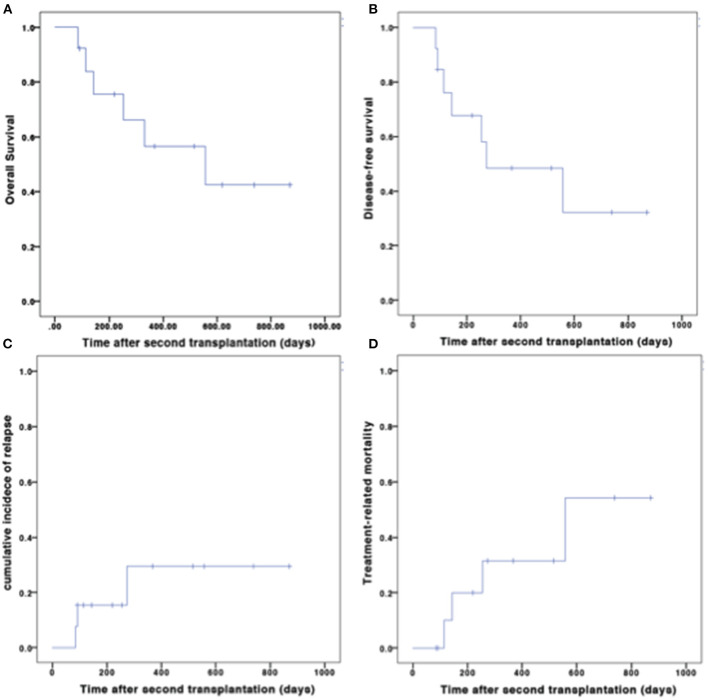
Outcomes after second transplantation. **(A)** OS; **(B)** DFS; **(C)** CIR; **(D)** TRM.

The median follow-up time for survivors was 515 (range 92–869) days after the second transplantation. At the last follow-up, seven patients were alive. Among the six deaths, four were due to TRM, and two were due to relapse. The OS and DFS at 1 year after the second transplantation were 56.6 and 48.4%, respectively (Figure 3).

## Discussion

A second transplantation is almost the only salvage method for graft failure. The key point to successful re-transplantation consists of several important factors, including the optimal conditioning regimen, the donor, and supportive treatment. Unfortunately, there is no standard method for salvage transplantation. In the current study, we demonstrated that a transplantation with a reduced-intensity fludarabine and cyclophosphamide (Flu/Cy) conditioning regimen from different HIDs provides a very successful salvage for patients with GF after stem cell transplantation. Previous reports of salvage haploidentical stem cell transplantation were of small patient populations and case series, and importantly, most were retrospective and heterogenous in terms of conditioning regimens and protocol (13–21). To the best of our knowledge, this is the first prospective study investigating salvage haploidentical transplantation in patients with GF.

Due to the urgent need for a second transplantation in patients with GF, fast preparation of the donor is critical in clinical practice. Obviously, it is not practical to search for unrelated volunteer donors from the registry. CB and HIDs are alternative choices. A recent Japanese study demonstrated the engraftment advantage of an HID over CB (22). Beyond that, the HID has several other advantages, such as wide availability of multiple donors and sufficient cell number. Therefore, haploidentical transplantation as a salvage for GF is a promising choice.

Because a second transplantation is usually performed soon after the first transplantation, reconditioning might lead to significant accumulative organ toxicities and TRM. It has been demonstrated that a reduced-intensity conditioning regimen is associated with favorable outcomes in previous reports (22, 23). Thus, while a reduced-intensity conditioning regimen is generally recommended for second transplantation, there is no standard conditioning regimen. Most previous reports utilize fludarabine- and/or total body irradiation (TBI)-based conditioning regimens (summarized in Table 3). The optimal conditioning regimen should maintain sufficient immunosuppressive effects on one hand to promote engraftment, while on the other hand it should have a reduced intensity to lessen the toxicity given that patients are very fragile soon after the first transplantation.

**Table 3:** Summary of reports utilizing haplo-SCT as salvage for graft failure.

**Year**	**Author**	**Country**	**Study**	***N***	**First donor**	**Disease**	**GF type**	**Time to 2SCT**	**Second conditioning**	**Second donor**	**Different donor**	**GVHD prophylaxis**	**Engraftment**	**aGVHD**	**cGVHD**	**Relapse**	**TRM**	**OS**	**DFS**
2012	Yoshihara et al. (13)	Japan	R	8	CB3, Haplo5	M, 9	pGF6, sGF2	36	FLU90THI10ATG2-5TBI2-4	Haplo	5/8	FK + MP	8/8	4/8	0	1/8	3/8	5/8	4/8
2012	Kanda et al. (19)	Japan	R	11	haplo6, CB2	M, 10; NM, 1			FLU30CY2ALE20TBI2	haplo19msd1	5	MMF + CNI	10/11	3/8	4/8		3/11	8/11	
2014	Moscardo et al. (15)	Spain	R	11	CB	M9, NM2	pGF7, sGF4	46	FLU150ATG8	Haplo	11	T cell depletion	7/11	1/7	2/6	2/7	6/11	3/11	
2015	Tang et al. (16)	China	R	17	CB	M 17	pGF17	38	FLU120ATG7.5CY50TBI3	Haplo	17	CSA + MMF	14/17	6/14	5/12		7/17	10/17	
2017	Mochizuk et al. (18)	Japan	R	6	CB4, Haplo2	AL 6	pGF	28-126	FLU90-140MEL140ATG	Haplo	5	FK + PRED + MTX	5/5	4/5	3/5		2/6	4/6	
2018	Kliman et al. (14)	Australia	R	5	CB1, Haplo2, URD2	M2, NM3	pGF2sGF3		FLU150CY29TBI2	Haplo		PTCy + MMF + FK	5/5	1/5			1/5	3/5	
2019	Wegenr et al. (20)	Germany	R	33		M25,NM8		22	TNI7 based	Haplo28	27	T cell depletion	32/33				22.3%	65.1%	
2019	Prata et al. (17)	France	R	24	CB	M20, NM4			Mainly Flu/Cy/TBI	Haplo	20	PTCy + CSA	79%	14%	31%		8/24	56%	
2020	Kongtim et al. (21)	USA	R	31	Haplo19, CB8	M31		48	Mainly Flu/Cy/TBI	Haplo	24	PTCy + MMF + FK	27/31	35.5	14.9	14.9%	59%	22%	
2020	Current study	China	P	13	URD2, Haplo11	M13	pGF12, sGF1		FLU/Cy	Haplo	All	CsA + MMF + basixilimab	13/13						

*R, retrospective; P, prospective; CB, cord blood; haplo, haploidentical; URD, unrelated donor; pGF, primary graft failure; sGF, secondary graft failure*.

It is encouraging that none of our patients had an early death due to regimen-related toxicity. Our conditioning regimen is less intense than the regimens used in previous reports. In addition, in our current regimen, we did not include TBI because to do so would mean leaving the protection of the laminar flow bed, increasing the risk of infection. Interestingly, although it seems to be less intense than almost all previous reports, the Flu/Cy regimen used in our study demonstrated very good performance in terms of fast engraftment. This suggests that the re-transplant conditioning regimen might not need to be so intense in the early period after the first transplantation as that might have already suppressed the recipient immune system to some extent. Our results indicate that the Flu/Cy regimen in our study meets the criteria of our previously defined optimal conditioning regimen. However, further studies are needed to confirm our preliminary encouraging results.

It is still unclear whether the patients should undergo a second transplantation from a different donor or the same donor. The results of previous reports are controversial (8). It is difficult to draw conclusions from previous studies because all these reports are retrospective studies with very small sample sizes and highly heterogeneous populations, transplant protocols, and donor type. The association of DSA with graft failure has been clearly demonstrated in mismatched unrelated donor transplantation, cord blood transplantation, and haploidentical transplantation. Therefore, transplantation for graft failure due to DSA should with a different donor. Furthermore, the mechanism of graft failure has not been fully elucidated, there might be some donor-recipient interaction beyond DSA. We hypothesize that a different HID with different mismatched haplotypes might be a preferred donor choice to avoid a second rejection, especially for those with suspected T-cell rejection as the cause of GF. In our study, we used a different donor, and this demonstrated very encouraging results. Recently, Kongtim et al. suggested that a second transplantation with the same donor has a high engraftment failure rate and TRM. In that study, only four out of the seven patients that received the same donor for the second transplantation, engrafted successfully. However, all seven of those patients died of NRM, while all of the patients with different donors engrafted successfully. Changing to a different donor might be one of the important features of a successful engraftment (21). Furthermore, using an initial donor in a very short period might increase the risk for re-collection with the initial donor. Considering that it is not possible to perform a randomized study to investigate the impact of different donors, re-transplantation with different HIDs might be a very practical method. However, this requires further validation.

It is interesting that the GVHD prophylaxis seems to be less intensive than that used for first haploidentical transplantation, while the GVHD incidence is acceptable. Patient retransplanted at early time after first transplantation might still at intensive immunosuppressed status. The possible reasons might include the poor reconstitution of hematopoiesis, and also the drugs used for first transplantation might still have impact (such as ATG which could be detected in serum up to 3-4 months after transplantation).

Although our regimen demonstrated excellent engraftment and an early safety profile, the GVHD and infection complications observed were still very high. Therefore, this protocol requires further investigation and optimization to reduce GVHD and infection which would translate into long-term survival.

In conclusion, this phase-2 prospective study demonstrated that a second transplantation with Flu/Cy conditioning from a different HID was a successful salvage for GF after first transplantation in patients with hematological malignancies.

## Data Availability Statement

The original contributions presented in the study are included in the article/Supplementary Material, further inquiries can be directed to the corresponding author/s.

## Ethics Statement

The studies involving human participants were reviewed and approved by Peking University People's Hospital. Written informed consent to participate in this study was provided by the participants' legal guardian/next of kin.

## Author Contributions

X-JH and Y-QS designed the study and prepared the manuscript. All authors approved the final manuscript and submission.

## Conflict of Interest

The authors declare that the research was conducted in the absence of any commercial or financial relationships that could be construed as a potential conflict of interest.

## References

[1] Olsson RF Logan BR Chaudhury S Zhu X Akpek G Bolwell BJ. Primary graft failure after myeloablative allogeneic hematopoietic cell transplantation for hematologic malignancies. Leukemia. (2015) 29:1754–62. 10.1038/leu.2015.75 25772027 PMC4527886

[2] Aversa F Terenzi A Tabilio A Falzetti F Carotti A Ballanti S. Full haplotype-mismatched hematopoietic stem-cell transplantation: a phase II study in patients with acute leukemia at high risk of relapse. J Clin Oncol. (2005) 23:3447–54. 10.1200/JCO.2005.09.117 15753458

[3] Luznik L O'Donnell PV Symons HJ Chen AR Leffell MS Zahurak M. HLA-haploidentical bone marrow transplantation for hematologic malignancies using nonmyeloablative conditioning and high-dose, posttransplantation cyclophosphamide. Biol Blood Marrow Transplantation. (2008) 14:641–50. 10.1016/j.bbmt.2008.03.005 18489989 PMC2633246

[4] Wang Y Liu DH Liu KY Xu LP Zhang XH Han W. Long-term follow-up of haploidentical hematopoietic stem cell transplantation without *in vitro* T cell depletion for the treatment of leukemia: nine years of experience at a single center. Cancer. (2013) 119:978–85. 10.1002/cncr.27761 23097265

[5] Olsson R Remberger M Schaffer M Berggren DM Svahn BM Mattsson J. Graft failure in the modern era of allogeneic hematopoietic SCT. Bone Marrow Transplantation. (2013) 48:537–43. 10.1038/bmt.2012.239 23222384

[6] Guardiola P Kuentz M Garban F Blaise D Reiffers J Attal M. Second early allogeneic stem cell transplantations for graft failure in acute leukaemia, chronic myeloid leukaemia and aplastic anaemia. Br J Haematol. (2000) 111:292–302. 10.1111/j.1365-2141.2000.02306.x 11091216

[7] Sun Y Xu L Liu D Zhang X Wang Y Han W. [Second transplantation for 22 patients with graft failure after first allogeneic stem cell transplantation]. Zhonghua Xue Ye Xue Za Zhi. (2014) 35:673–7. 10.3760/cma.j.issn.0253-2727.2014.08.001 25152110

[8] Ferra C Sanz J Diaz-Perez MA Morgades M Gayoso J Cabrera JR. Outcome of graft failure after allogeneic stem cell transplant: study of 89 patients. Leukemia Lymphoma. (2015) 56:656–62. 10.3109/10428194.2014.930849 24913510

[9] Przepiorka D Weisdorf D Martin P Klingemann HG Beatty P Hows J. 1994 consensus conference on acute GVHD grading. Bone Marrow Transplantation. (1995) 15:825–8. 7581076

[10] Sullivan KM Agura E Anasetti C Appelbaum F Badger C Bearman S. Chronic graft-versus-host disease and other late complications of bone marrow transplantation. Seminars Hematol. (1991) 28:250–9. 1887253

[11] Sorror ML Maris MB Storb R Baron F Sandmaier BM Maloney DG. Hematopoietic cell transplantation (HCT)-specific comorbidity index: a new tool for risk assessment before allogeneic HCT. Blood. (2005) 106:2912–9. 10.1182/blood-2005-05-2004 15994282 PMC1895304

[12] Chang YJ Xu LP Wang Y Zhang XH Chen H Chen YH. Rituximab for desensitization during HLA-mismatched stem cell transplantation in patients with a positive donor-specific anti-HLA antibody. Bone Marrow Transplantation. (2020) 55:1326–36. 10.1038/s41409-020-0928-z 32385341

[13] Yoshihara S Ikegame K Taniguchi K Kaida K Kim EH Nakata J. Salvage haploidentical transplantation for graft failure using reduced-intensity conditioning. Bone Marrow Transplantation. (2012) 47:369–73. 10.1038/bmt.2011.84 21478920

[14] Kliman D Bilmon I Kwan J Blyth E Micklethwaite K Panicker S. Rescue haploidentical peripheral blood stem cell transplantation for engraftment failure: a single-centre case series. Int Med J. (2018) 48:988–91. 10.1111/imj.13979 30133987

[15] Moscardo F Romero S Sanz J Sanz MA Montesinos P Lorenzo I. T cell-depleted related HLA-mismatched peripheral blood stem cell transplantation as salvage therapy for graft failure after single unit unrelated donor umbilical cord blood transplantation. Biol Blood Marrow Transplantation. (2014) 20:1060–3. 10.1016/j.bbmt.2014.03.024 24685578

[16] Tang BL Zhu XY Zheng CC Liu HL Geng LQ Wang XB. Successful early unmanipulated haploidentical transplantation with reduced-intensity conditioning for primary graft failure after cord blood transplantation in hematologic malignancy patients. Bone Marrow Transplantation. (2015) 50:248–52. 10.1038/bmt.2014.250 25365067

[17] Prata PH Resche-Rigon M Blaise D Socie G Rohrlich PS Milpied N. Outcomes of salvage haploidentical transplant with post-transplant cyclophosphamide for rescuing graft failure patients: a report on behalf of the francophone society of bone marrow transplantation and cellular therapy. Biol Blood Marrow Transplantation. (2019) 25:1798–802. 10.1016/j.bbmt.2019.05.013 31129355

[18] Mochizuki K Sano H Akaihata M Kobayashi S Waragai T Ohara Y. T cell replete-haploidentical second hematopoietic stem cell transplantation for primary graft failure in pediatric patients with hematologic malignancies. Pediatric Transplantation. (2017) 21. 10.1111/petr.13043 28845543

[19] Kanda J Horwitz ME Long GD Gasparetto C Sullivan KM Chute JP. Outcomes of a 1-day nonmyeloablative salvage regimen for patients with primary graft failure after allogeneic hematopoietic cell transplantation. Bone Marrow Transplantation. (2012) 47:700–5. 10.1038/bmt.2011.158 21804612 PMC3214602

[20] Wegener D Lang P Paulsen F Weidner N Zips D Ebinger M. Immunosuppressive total nodal irradiation-based reconditioning regimens after graft rejection or graft failure in pediatric patients treated with myeloablative allogeneic hematopoietic cell transplantation. Int J Radiation Oncol Biol Phys. (2019) 104:137–43. 10.1016/j.ijrobp.2018.12.031 30593907

[21] Kongtim P Bittencourt M Srour SA Ramdial J Rondon G Chen J. Haploidentical transplants for patients with graft failure after the first allograft. Am J Hematol. (2020). 10.1002/ajh.25917. [Epub ahead of print]. 32602112

[22] Harada K Fuji S Seo S Kanda J Ueki T Kimura F. Comparison of the outcomes after haploidentical and cord blood salvage transplantations for graft failure following allogeneic hematopoietic stem cell transplantation. Bone Marrow Transplantation. (2020) 55:1784–95. 10.1038/s41409-020-0821-9 32051535

[23] Grandage VL Cornish JM Pamphilon DH Potter MN Steward CG Oakhill A. Second allogeneic bone marrow transplants from unrelated donors for graft failure following initial unrelated donor bone marrow transplantation. Bone Marrow Transplantation. (1998) 21:687–90. 10.1038/sj.bmt.1701146 9578308

