# Supplementary material for: Graft Failure in Patients With Hematological Malignancies: A Successful Salvage With a Second Transplantation From a Different Haploidentical Donor
Source: Front Med (Lausanne). 2021 Jun 4;8:604085. doi: 10.3389/fmed.2021.604085 (PMC8212968; doi:10.3389/fmed.2021.604085)
Supplement: Supplementary file 1 [file Table_1.DOCX]

**Table S1. Acute GVHD after 2^nd^ transplantation**

| **ID** | **age** | **sex** | **disease** | **aGVHD**  **grade** | **aGVHD**  **organ** | **onset time**  **(days after 2nd transplantation)** | **first-line therapy** | **response** | **seconde-line therapy** | **response** |
| --- | --- | --- | --- | --- | --- | --- | --- | --- | --- | --- |
| 1 | 37 | male | AML | 1 | skin | 24 | MP | CR |  |  |
| 2 | 42 | female | AML | 0 |  |  |  |  |  |  |
| 3 | 33 | female | AML | 1 | skin | 12 | MP | CR |  |  |
| 4 | 8 | female | ALL | 0 |  |  |  |  |  |  |
| 5 | 55 | female | AML | 4 | skin | 27 | MP | PR | Basiliximab | CR |
| 6 | 37 | male | MDS | 4 | skin, liver, gut | 153 | MP | PD | Basiliximab | CR |
| 7 | 19 | male | ALL | 1 | skin | 88 | MP | CR |  |  |
| 8 | 21 | male | ALL | 4 | liver,gut | 40 | MP | PD | Basiliximab | CR |
| 9 | 34 | female | ALL | 0 |  |  |  |  |  |  |
| 10 | 37 | female | MDS | 2 | skin | 21 | MP | CR |  |  |
| 11 | 46 | female | AML | 2 | skin | 21 | MP | PR | Basiliximab | CR |
| 12 | 17 | male | NHL | 0 |  |  |  |  |  |  |
| 13 | 41 | female | ALL | 2 | skin | 54 | MP | CR |  |  |

Abbreviations: AML, acute myeloid leukemia; ALL, acute lymphoblastic leukemia; MDS, myelodysplastic syndrome;NHL, non-Hodgkin lymphoma; MP, Methylprednisolone； CR, complete remission; PR, partial remission; PD, progression disease
