# Supplementary figures and images for: Graft Failure in Patients With Hematological Malignancies: A Successful Salvage With a Second Transplantation From a Different Haploidentical Donor
Source: Front Med (Lausanne). 2021 Jun 4;8:604085. doi: 10.3389/fmed.2021.604085 (PMC8212968; doi:10.3389/fmed.2021.604085)

## Slide 1
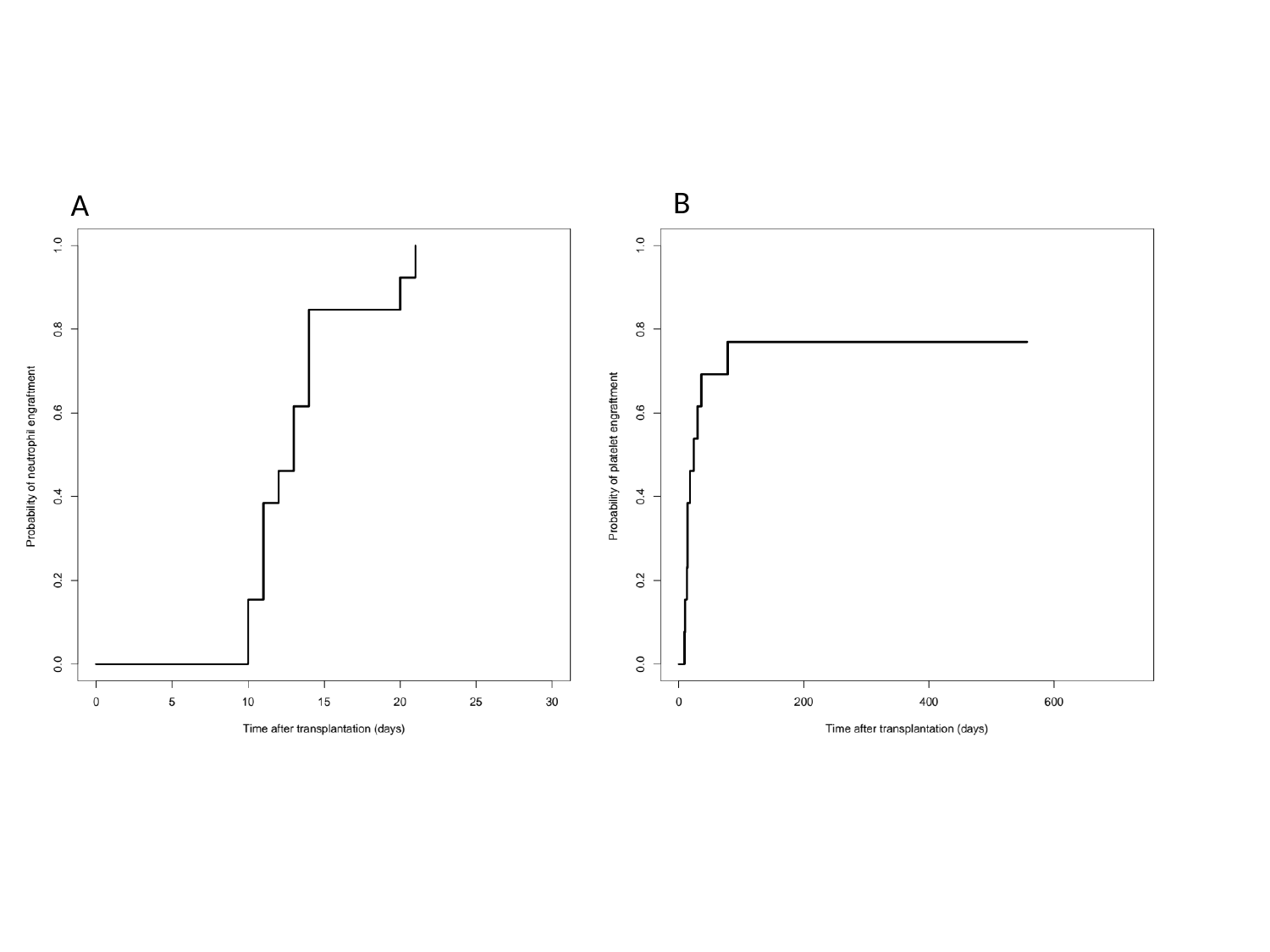

B
A

Supplement: Supplementary file 2 [file Presentation_1.PPTX]

## Slide 1
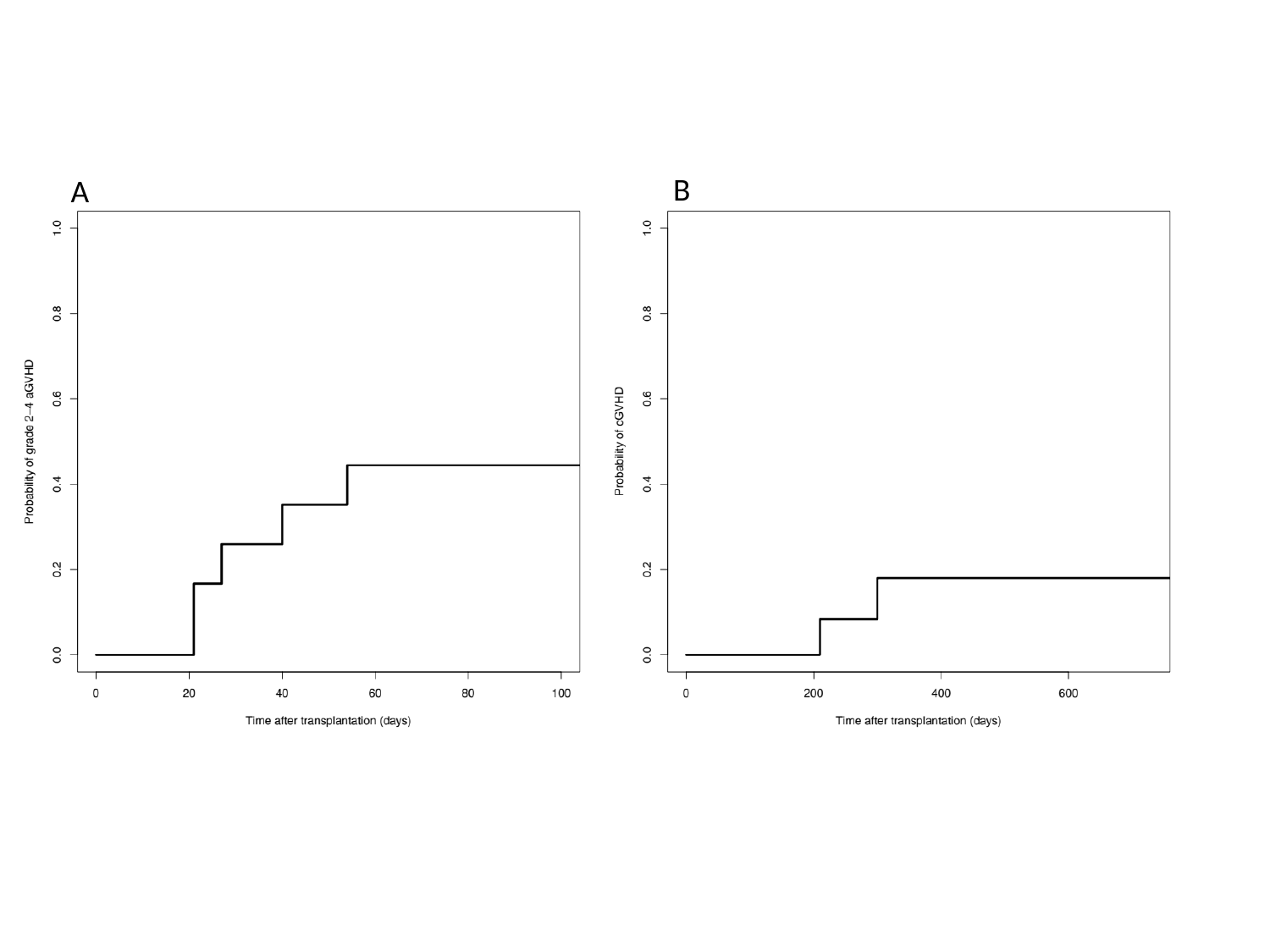

B
A

Supplement: Supplementary file 3 [file Presentation_2.PPTX]

## Slide 1
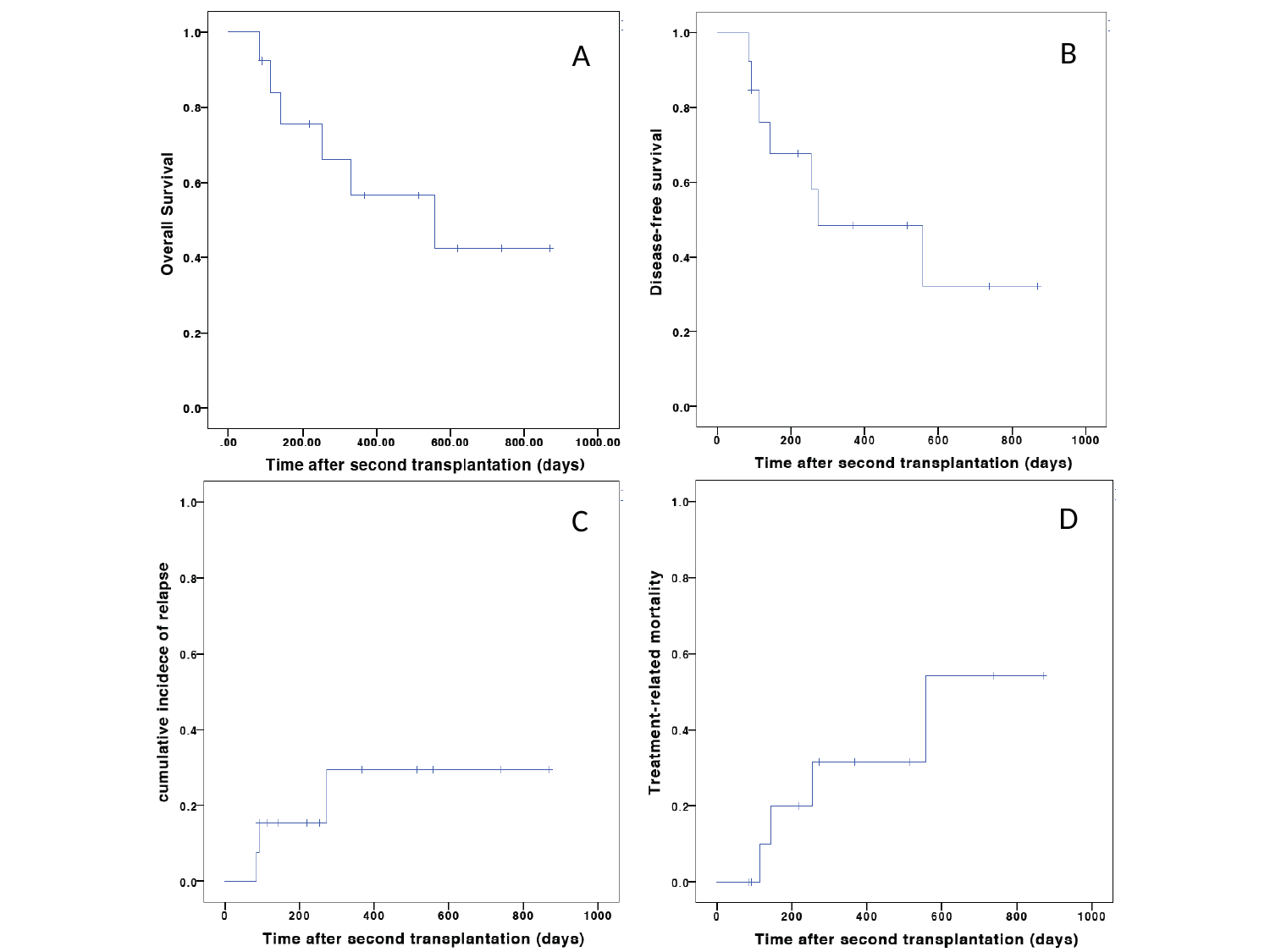

B
A
D
C

Supplement: Supplementary file 4 [file Presentation_3.PPTX]
